# Society for Cardiovascular Magnetic Resonance perspective on the ACC/AHA/ASE/ASNC/ASPC/HFSA/HRS/SCAI/SCCT/SCMR/STS 2023 multi-modality appropriate use criteria for the detection and risk assessment of chronic coronary disease

**DOI:** 10.1186/s12968-023-00959-4

**Published:** 2023-10-19

**Authors:** W. Patricia Bandettini, Raymond Y. Kwong, Amit R. Patel, Sven Plein, the Society for Cardiovascular Magnetic Resonance

**Affiliations:** 1 National Heart, Lung, and Blood Institute, National Institutes of Health, Bethesda, MD USA; 2 Cardiovascular Division, Department of Medicine, Brigham and Women’s Hospital, Boston, MA USA; 3 Cardiovascular Division, Department of Medicine, University of Virginia, Charlottesville, VA USA; 4 Leeds Institute of Cardiovascular and Metabolic Medicine, University of Leeds, Leeds, UK

**Keywords:** Cardiovascular magnetic resonance imaging, Society for Cardiovascular Magnetic Resonance, Chronic coronary disease, Chest pain, Ischemia, Risk assessment, Appropriate use, Guideline

## Background

The American College of Cardiology, together with other specialty and subspecialty societies, has recently published Appropriate Use Criteria (AUC) for the Detection and Risk Assessment of Chronic Coronary Disease (CCD) [1]. The document updates the 2013 AUC for the management of Stable Ischemic Heart Disease (SIHD) [2] and covers the use of radionuclide imaging, stress echocardiography (echo), coronary computed tomography angiography (CCTA) and calcium scoring, stress cardiovascular magnetic resonance (CMR), and invasive coronary angiography. The document aims to complement clinical practice guidelines and aid clinicians in decision-making for common clinical scenarios in CCD and implement best practices in patient care. Recommendations are given for symptomatic and asymptomatic patients with a spectrum of scenarios in each of the two categories.

The Society for Cardiovascular Magnetic Resonance (SCMR) was represented on the writing and rating panels of this document and approved its final version. Here we discuss the recommendations in the AUC from the perspective of the CMR practitioner and in the context of other relevant guidance.

### Changes from the 2013 AUC for multimodality imaging in SIHD

In the last decade, numerous studies, trials, and meta-analyses have established the diagnostic accuracy, cost-effectiveness, and predictive value of stress perfusion CMR in patients with CCD. In recognition of this increasing evidence for CMR, the ratings for stress CMR in the 2023 AUC for Multimodality Imaging in CCD have generally increased compared with the 2013 AUC for Multimodality Imaging in SIHD. The AUC classes of stress CMR are now equivalent to nuclear imaging and stress echocardiography across almost all clinical scenarios. The document recommends that where more than one test is rated as ‘appropriate’ in a clinical scenario, clinician judgment, test advantages and disadvantages, and local expertise should govern the choice of test for an individual patient. This recommendation now allows practitioners to choose stress CMR as the first line non-invasive functional imaging modality across a range of presentations of CCD.

The most recent AUC also includes a new category of ‘No Test’, and indeed is the first document that provided this AUC rating option across all the described clinical scenarios in CCD. This is an important addition because most patients presenting with stable chest pain are at low risk of adverse cardiac events. This addition provides useful guidance to clinicians when it may be in the best interest of the patient not to undergo further assessment.

Also of note, in addition to clinical pre-test probability assessment, the 2023 AUC document incorporates targeted patient clinical symptoms, scenarios, atherosclerotic cardiovascular disease risk assessment, and other factors that were not previously emphasized.

### The 2023 AUC for multimodality imaging in CCD in the context of other guidelines

The 2023 update brings the recommendations for the appropriate use of stress CMR more in line with other international practice guidelines. Both the 2021 AHA/ACC/ASE/CHEST/SAEM/SCCT/SCMR Guideline for the Evaluation and Diagnosis of Chest Pain [3] and the 2019 ESC Guidelines for the Diagnosis and Management of Chronic Coronary Syndromes [4] generally rate stress CMR, nuclear imaging and stress echocardiography with the same level of indication, including Class 1 indications in many clinical scenarios.

However, some differences with these practice guidelines remain. In the 2021 US chest pain guidelines [3], CMR and positron emission tomography (PET) have an additional Class 2a indication for the quantification of myocardial blood flow reserve (MBFR) to improve diagnostic accuracy and enhance risk stratification and to detect coronary microvascular disease. Similarly, the 2019 ESC guidelines assign transthoracic Doppler of the left anterior descending artery, stress CMR, and PET a Class 2b recommendation for assessing patients with suspected microvascular disease [4]. The 2023 AUC document does not specifically cover coronary microvascular disease and includes only a ‘may be appropriate’ rating for PET and CMR in patients with normal anatomical coronary imaging, which may be applicable to patients with suspected coronary microvascular disease.

Importantly, the 2023 AUC guidelines also do not specifically address sex as a biological variable, while the 2021 US chest pain guidelines mention “the uniqueness of chest pain in women”. CMR may be particularly suited for the assessment of women with CCD as it does not expose patients to ionizing radiation, has a higher spatial resolution for smaller-sized hearts than nuclear imaging, and can quantify myocardial blood flow in women with chest pain but no obstructive coronary disease.

### Comparison of imaging modalities

Consistent with the process of previous AUC development, only the median score from the rating panel was used in formulating the AUC class as “appropriate” (A), “may be appropriate” (M), or “rarely appropriate” (R) in each of the 64 described clinical scenarios. The AUC process aims to collect inputs from a broad array of stakeholders with variable experience in the different diagnostic tests. These rating panel members were presented with a description of the current literature relevant to each of the clinical scenarios and were asked to score the appropriateness of each modality based on clinical impact, safety, and cost. Stress CMR remains a modality less familiar in practice to some of the rating panel members, compared to the other modalities, and this likely prohibited CMR in achieving higher rating scores in some of the scenarios.

Notably, the AUC specifically refrained from competitive ranking of imaging modalities, citing 'the limited availability of comparative evidence, patient variability, and the range of capabilities available in any given local setting’. This approach aims to provide general evidence of each modality toward clinical use, but it neglects the growing body of evidence that shows higher diagnostic accuracy and effectiveness of management of PET and CMR over SPECT and stress echocardiography to detect significant coronary artery stenosis. Stress CMR has been compared with SPECT in single-center and multi-center studies, which have consistently shown higher diagnostic accuracy over SPECT [5, 6] and comparable diagnostic performance of CMR against PET [7, 8]. While less evidence exists for their direct comparison, meta-analyses have indicated higher performance of CMR against stress echocardiography [7, 8].

The AUC also has not provided specific guidance on the cost-effectiveness of the different imaging modalities—an important factor in clinical practice, particularly when multiple tests are available. Within a group of stable chest pain patients in the US, stress CMR was a cost-effective gatekeeping tool, and its use avoids unnecessary invasive coronary angiography [9]. Similarly, within the UK, the CE-MARC study performed a cost-analysis of different strategies that included different combinations of exercise stress testing, SPECT, CMR, and coronary angiography, and found that the two most cost-effective strategies were ones that utilized CMR [10].

Instead of competitive rankings, the AUC encourage physicians to consider patient-specific and local factors in choosing an appropriate test. To aid this decision-making process, the document includes sections listing the ‘advantages’ and ‘limitations’ of the different tests. For CMR, the advantages are listed as: “Can assess wall motion, ischemia, and infarction in one study. Can quantify myocardial blood flow to improve test accuracy and assess myocardial and pericardial diseases. Can perform viability testing.” A key advantage of stress CMR that may have also warranted a mention is its unique ability to provide a co-registered assessment of cardiac structure/function, ischemia, and tissue characterization, which accurately quantifies ischemia and infarct burden at both global and segmental levels. In addition to characterizing ischemia/viability from coronary disease, this multi-parametric capability of CMR can help simultaneously diagnose other causes of chest pain and cardiac symptoms. In particular, myocarditis and pericarditis may not be detectable using some of the other imaging modalities but are readily identifiable on CMR and their inflammatory states can be quantified to monitor disease progression. Hypertrophic cardiomyopathy and other cardiomyopathies, such as arrhythmogenic cardiomyopathy (ACM) which may require clear visualization of subtle abnormal anatomy, are better assessed using CMR, which has high spatial resolution and is not limited by imaging windows. During a standard CMR, at least a qualitative assessment of valvular heart disease is also routinely made, allowing, for example, the detection of significant aortic stenosis as a potential cause of chest pain.

The ‘limitations’ of CMR are listed in Table A of the AUC document as ‘Claustrophobia, artifacts, and safety precautions with metallic medical devices’. However, in clinical routine, claustrophobia can usually be overcome with sedation or the use of wide bore MRI systems, and artifacts do not affect CMR more than other imaging modalities. Additionally, technical development of CMR has progressed to the extent of being able to mitigate a majority of common artifacts related to irregular heart rhythms and breathing. And lastly, specific processes exist for CMR to enable diagnostic quality imaging in patients with metallic devices, and the vast majority of pacemakers, implantable cardioverter defibrillators, and other implantable cardiac monitors (e.g. loop recorder) as well as metallic heart valves are no longer contraindicated for CMR [11, 12].

Table B of the AUC lists “Examples of Inconclusive Stress Imaging” and for CMR, “artifacts and arrhythmia”. In reality, with the current technology available and appropriate operator training, inconclusive stress CMR related to artifacts and arrhythmias is rare; inadequate vasodilator response is more common but can often be identified by assessment of splenic switch-off for adenosine stress or quantitative perfusion and is a limitation of all pharmacologic stress testing modalities.

Overall, CMR will be an excellent, and often the preferred choice for the assessment of CCD, when multiple tests are available locally. CMR offers the most comprehensive assessment of both coronary disease and other causes of chest pain in a single examination and is a test that is free of ionizing radiation and with high diagnostic accuracy.

## Comments on specific indications

### 1. Symptomatic patients

Within the 'symptomatic patients with no known CCD and no prior testing' section (Table 1.1), treadmill ECG or exercise stress testing (EST) scored higher or equal to imaging in 4 out of 5 scenarios despite its known reduced diagnostic accuracy. Note that the scores for EST were high in the low-likelihood ischemia groups, but as the likelihood of CAD increased (i.e., the symptoms seemed more convincing for CCD), the imaging modality scores increased over those of EST.

**Table 1.1:**
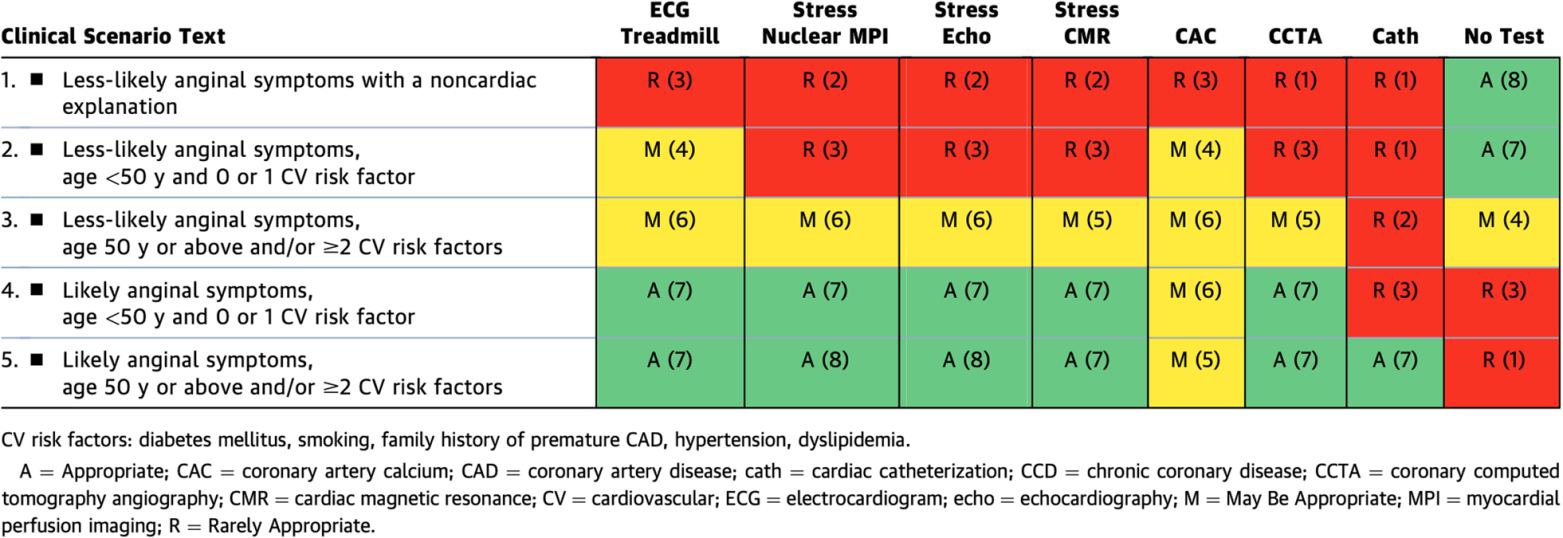
Symptomatic Patients With No Known CCD and No Prior Testing

In scenario 3, in individuals with two or more cardiovascular risk factors who have chest pain less likely to represent angina, stress CMR is categorized as “may be appropriate”. This recommendation is similar to stress nuclear imaging, stress echo, and coronary CTA; however, this grading underestimates the value of CMR in evaluating non-ischemic causes of chest pain that is widely recognized by experienced practitioners of CMR and highlights the need for efforts to increase awareness of the advantages of stress CMR.

There was remarkable consistency in scoring among the stress nuclear imaging, stress echo, and stress CMR categorization in the ‘symptomatic without known CCD’ and ‘with prior testing’ scenarios (Table 1.2). It is interesting to note one category in which CMR scored higher than stress nuclear imaging and echo: “Invasive coronary angiography with mild or no CAD and/or normal invasive physiologic testing”. This specific rating reflects the ability of CMR to diagnose microvascular disease and other disease states outside of obstructive epicardial coronary artery disease, such as myopericarditis.

**Table 1.2:**
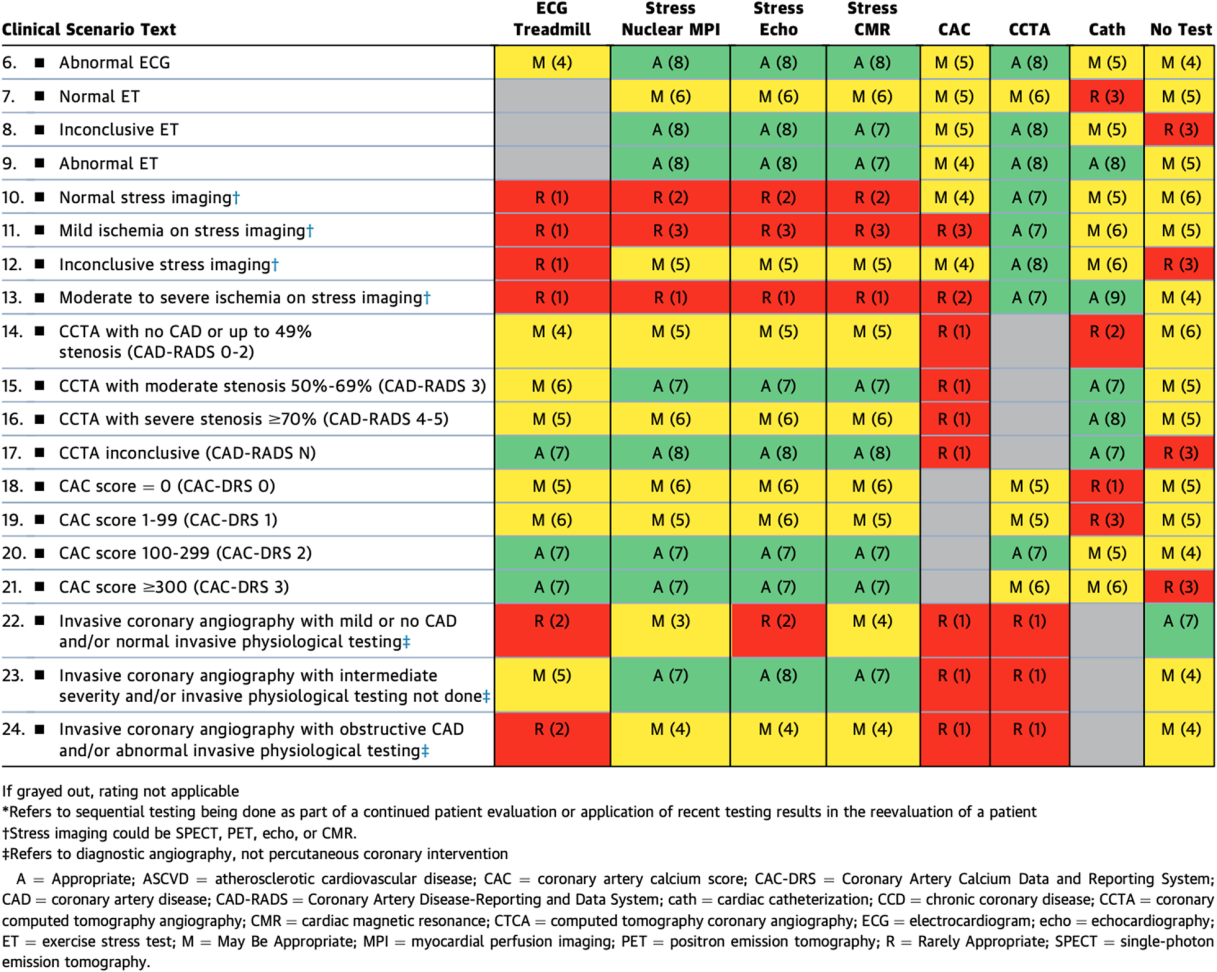
Symptomatic Patients Without Known CCD and With Prior Testing

Another scenario within ‘the symptomatic without known CCD but with prior testing’ group is that of the CAC score ≥ 300, in which all stress modalities were categorized as ‘appropriate’, whereas CCTA was ranked slightly lower as ‘may be appropriate’.

While stress CMR was rated similarly to the other stress imaging modalities in the category of ‘symptomatic patients with prior MI/revascularization’ (Table 1.3), this 2023 rating represents a higher, more supportive rating for CMR than the older consensus guidelines. In previous guidelines, CMR received a ‘may be appropriate’ rating for the indication of ‘obstructive CAD on invasive coronary angiography’ compared to exercise ECG, stress nuclear imaging, and stress echo methods that all were rated as ‘appropriate’. This reflects the increasing awareness and clinical evidence of the utility of CMR in these scenarios.

**Table 1.3:**
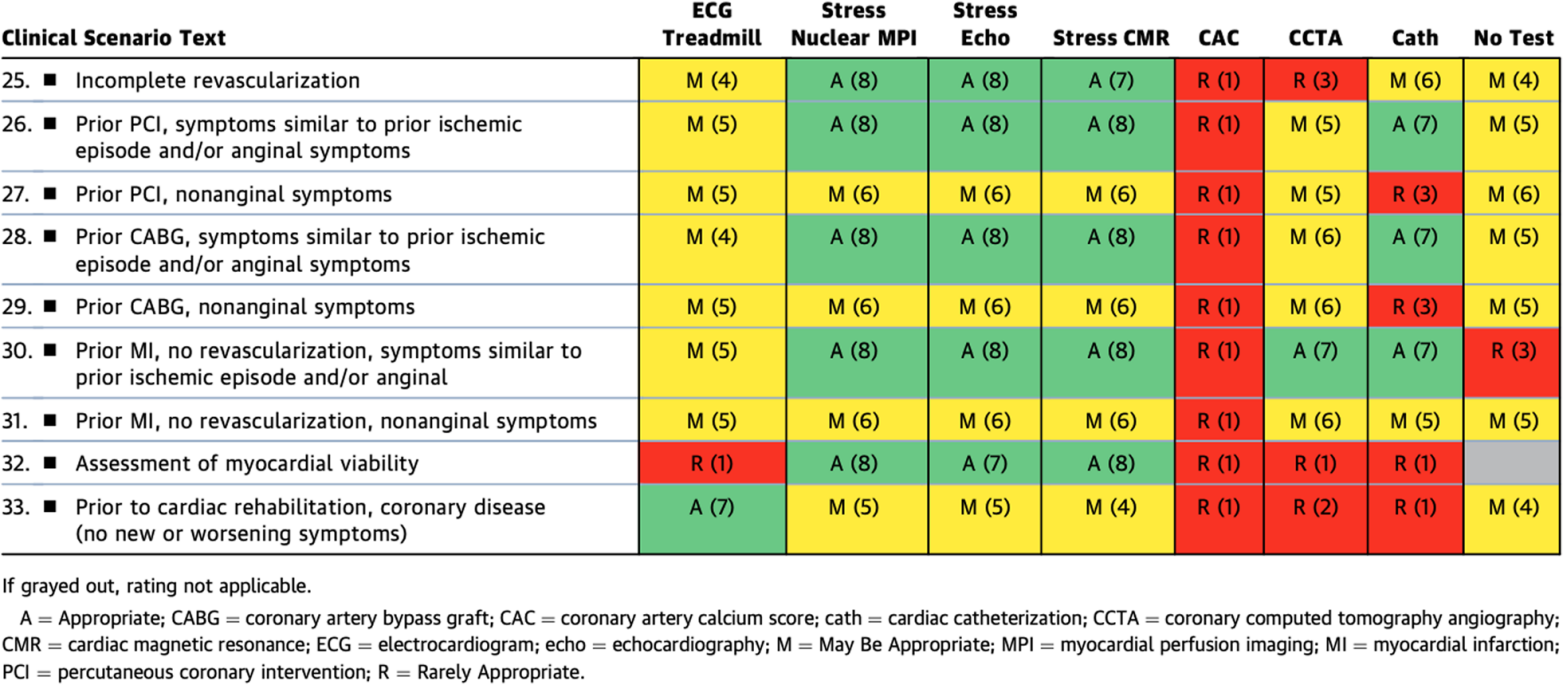
Symptomatic Patients With Prior MI or Revascularization

### 2. Asymptomatic patients

Not surprisingly, within the ‘asymptomatic patients with no known ASCVD’ group (Table 2.1), none of the stress imaging modalities were considered appropriate in the low to intermediate ASCVD risk categories, but there was again a uniform rating of ‘might be appropriate’ for the stress test modalities in patients with high (> 20%) ASCVD risk. In considering which patients might have higher risk, one must include patients with diabetes mellitus and patients with systemic inflammatory conditions, such as systemic lupus erythematosus (SLE), rheumatoid arthritis (RA), and human immunodeficiency virus (HIV) [13]. While CMR was rated comparably to the other modalities, CMR’s additional strengths in identifying subclinical myocardial infarction, fibrosis, and myocardial inflammation are not reflected in the rating, which solely focuses upon the ischemic assessment, but should be considered in clinical practice.

**Table 2.1:**
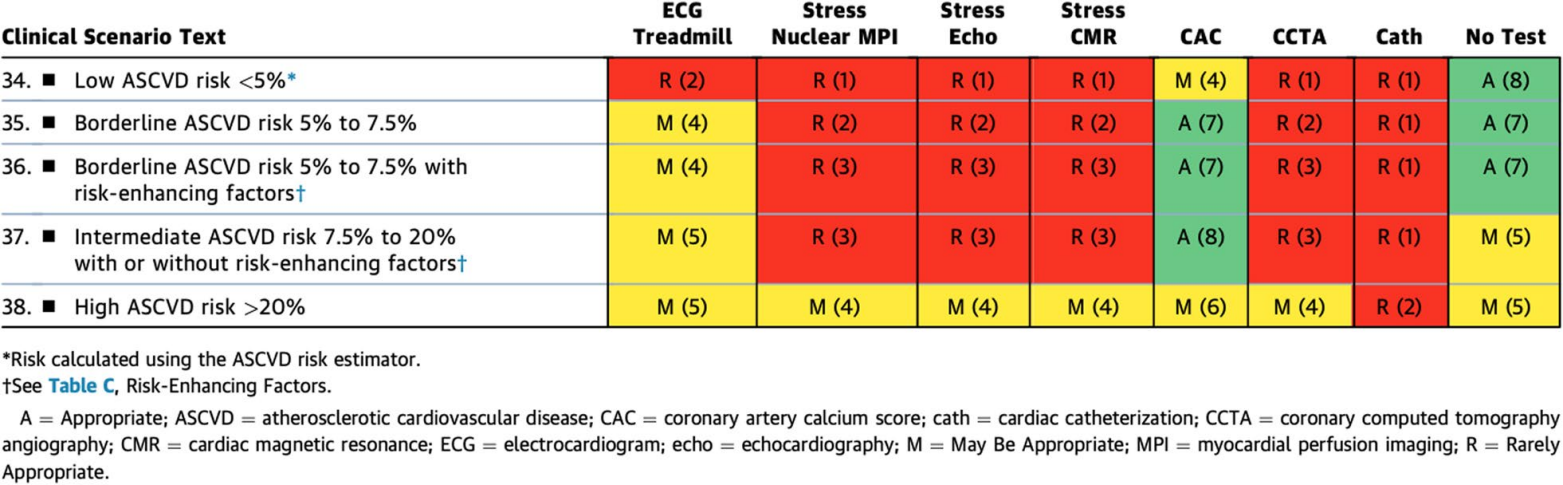
Asymptomatic Patients Without Known ASCVD

Similarly, within the ‘asymptomatic patients with prior revascularization or MI’ (Table 2.2), there was almost exact consensus among stress nuclear imaging, stress echo, and stress CMR for the evaluation of asymptomatic patients with prior revascularization or MI. The stress imaging modalities were deemed 'appropriate' for use in patients at high risk for or with a history of silent ischemia. Stress CMR and stress nuclear imaging both were rated 'appropriate' for assessment of myocardial viability over stress echo, which was rated as ‘might be appropriate’. It is worth emphasizing that CMR has higher spatial resolution over nuclear techniques for the detection of myocardial scar, allowing better identification of subendocardial MI, so may be the preferred test in patients with known MI [14, 15].

**Table 2.2:**
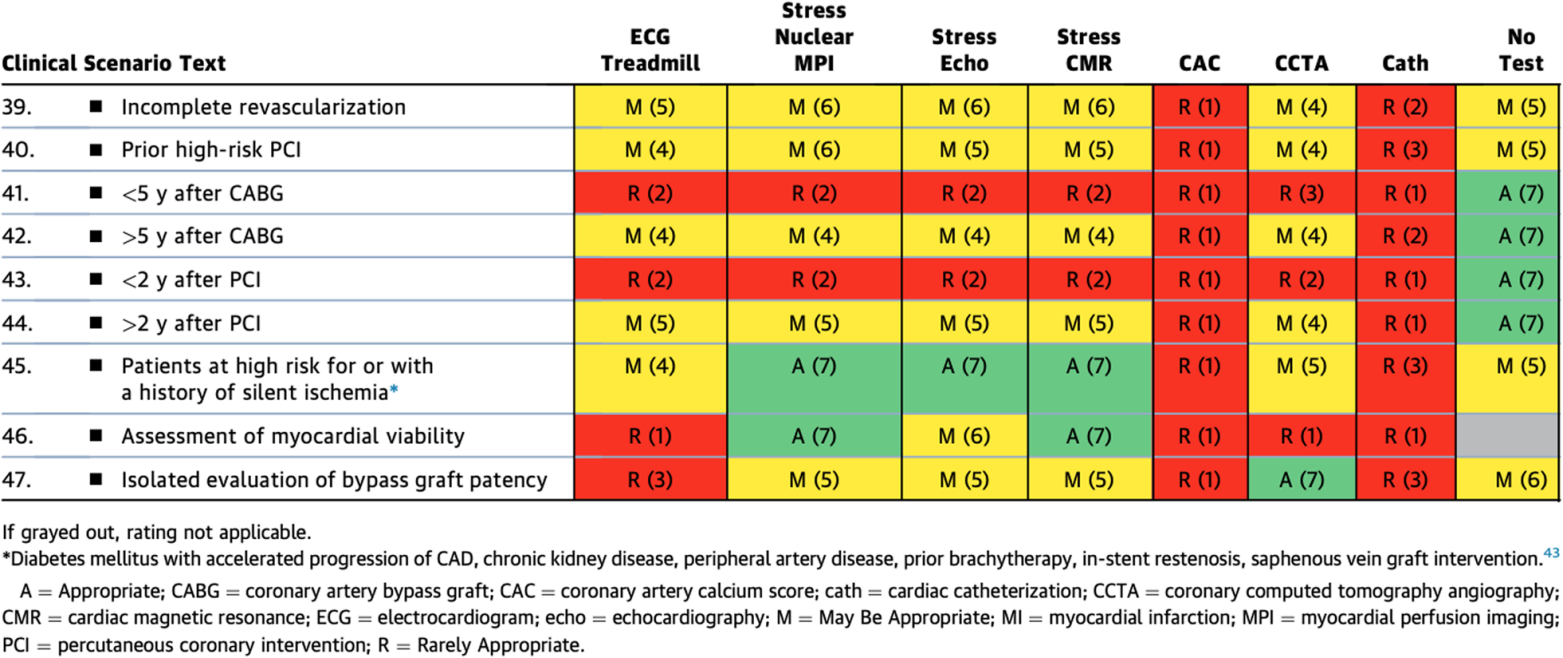
Asymptomatic Patients With Prior Revascularization or MI

In 'asymptomatic patients undergoing assessment prior to an exercise program or cardiac rehabilitation' (Table 2.3), exercise ECG was the only ‘appropriate’ test for patients with known CCD. All three stress imaging modalities were rated ‘may be appropriate’ for the same category; however, the ability to quantify myocardial scar by either CMR or nuclear imaging was not highlighted. Specifically, independently of assessing for myocardial ischemia, CMR offers the ability to aid in predicting arrhythmic risk using even basic left ventricular ejection fraction and quantification of MI [16].

**Table 2.3:**
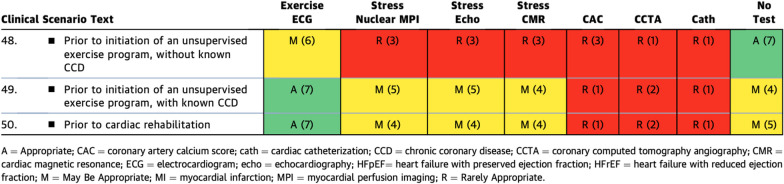
Asymptomatic Patients Undergoing Assessment of an Exercise Program or Cardiac Rehabilitation

Within the assessment of ‘other cardiovascular conditions in patients without signs of ischemia’ (Table 2.4), overall, CMR was rated similarly to stress echo and stress nuclear for most categories. All three stress imaging modalities were considered ‘appropriate’ for evaluation of heart failure—both reduced ejection fraction and preserved ejection fraction variants—as well as when evaluating patients with ventricular arrhythmia. CMR may have warranted higher scores for the assessment of myocardial inflammation, heart failure and arrhythmia (i.e., both atrial and ventricular) diagnoses because of its ability to non-invasively detect inflammation, clearly phenotype cardiomyopathy and provide risk assessment in arrhythmia; however, the focus of the consensus guidelines was on CCD with an emphasis on assessments of myocardial ischemia or coronary anatomy.

**Table 2.4:**
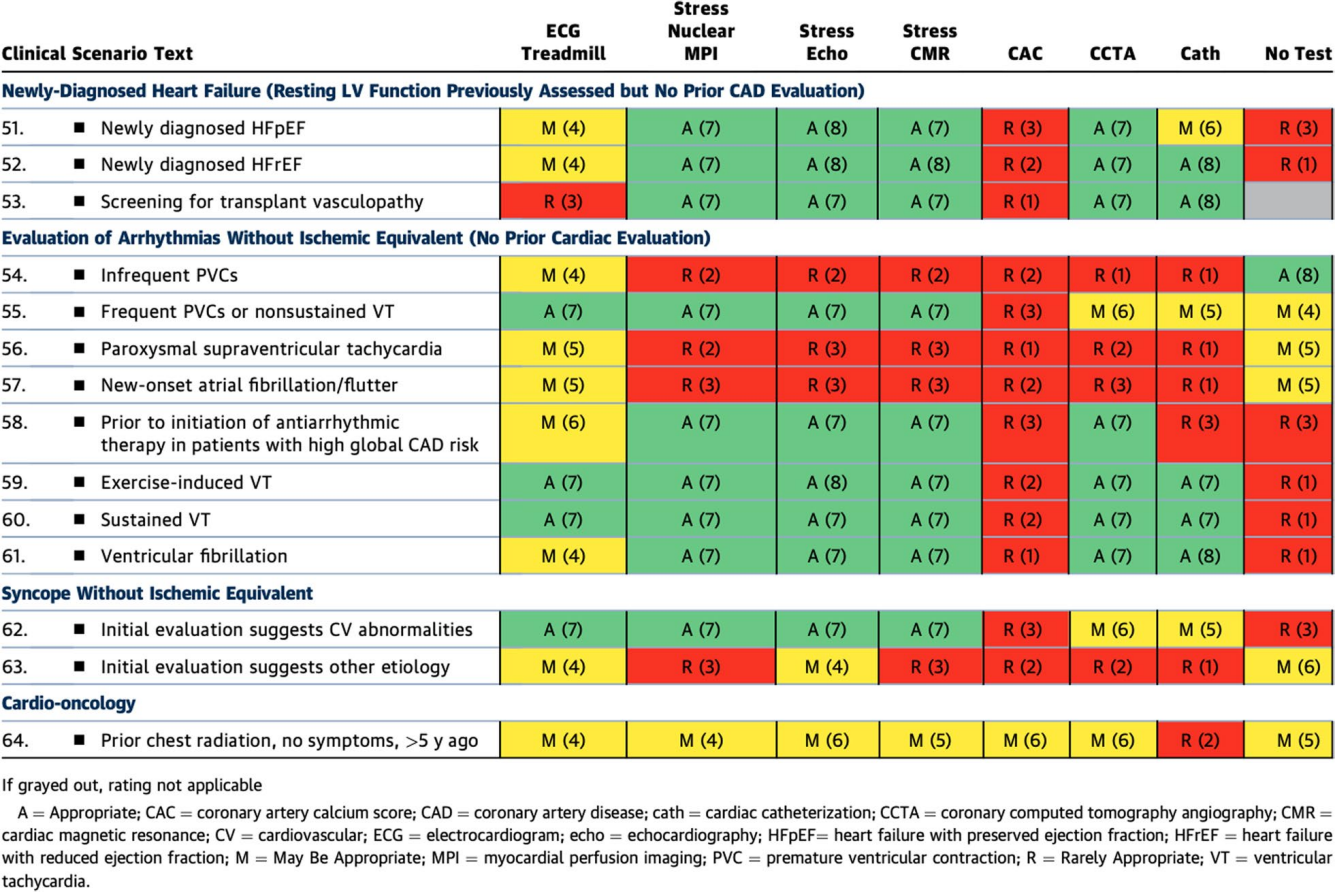
Other Cardiovascular Conditions in Patients Without Symptoms of Ischemia

For unclear reasons, for the indication of syncope without ischemic equivalent with an “other etiology” suggested beyond that of cardiovascular diagnoses, both stress CMR and stress nuclear were rated as ‘rarely appropriate’ while stress echo was rated as ‘might be appropriate’. These ratings seem at odds with clinical practice and practical reasoning as both echocardiography and CMR provide structural heart information.

Similarly, the ‘might be appropriate’ rating of CMR (and the other stress imaging modalities) in the cardio-oncology category and indication of prior chest radiation with no symptoms is more tentative than CMR clinical practice seems to indicate in cardio-oncology patients; however, this middle-ground rating likely relates to the fact that these AUC are focused upon CCD. Thus, CMR’s versatility in evaluating the cardio-oncology patient’s overall cardiovascular health is not expressed within the rating listed here. Stress and rest CMR are highly useful in assessing cardiomyopathy, myocarditis, pericarditis, valvular heart disease, and chest pain syndromes in the cardio-oncology patient [17].

## Conclusions

In the ACC/AHA/ASE/ASNC/ASPC/HFSA/HRS/SCAI/SCCT/SCMR/STS 2023 multi-modality appropriate use criteria for the detection and risk assessment of chronic coronary disease, CMR is now rated as comparable against the other imaging tools in almost all clinical scenarios. This rating reflects the substantially increased evidence for and awareness of CMR, compared to the last AUC document in 2013. Furthermore, the utility of CMR in the evaluation of nonobstructive epicardial CAD, i.e., microvascular disease, is recognized in the document. The unique ability of tissue characterization with CMR and the comprehensive ability of CMR to characterize myocardial physiology and health are not fully represented in this AUC document focused on CCD but are major advantages of CMR in evaluating patients with chest pain, heart failure, and arrhythmia, especially for non-ischemic etiologies.

Many of the 'limitations' of CMR that are listed in the document are either addressable or not a significant issue with the constant development of MRI technology. A comprehensive CMR exam in chronic CAD and heart failure can be completed in under 30 min without radiation or complex post-processing [18]; thus, accessibility to and usage of stress CMR should continue to grow. It is a charge to the CMR community, administrators and health care policy makers to remove remaining barriers to CMR access and payor coverage. Additionally, CMR education needs continued expansion and affirmation within all of those referring to and utilizing CMR.

The overall improved ‘appropriateness’ of CMR in the 2023 iteration of the AUC reflects positive progress in validating and disseminating CMR in clinical practice. Continued advocacy of CMR in daily cardiovascular care must be pronounced and steadfast.

## Data Availability

Not applicable.

## References

[1] Winchester DE, Maron DJ, Blankstein R, Chang IC, Kirtane AJ, Kwong RY, et al. ACC/AHA/ASE/ASNC/ASPC/HFSA/HRS/SCAI/SCCT/SCMR/STS 2023 multimodality appropriate use criteria for the detection and risk assessment of chronic coronary disease. J Am Coll Cardiol. 2023;81(25):2445–67. 10.1016/j.jacc.2023.03.410 37245131

[2] Wolk MJ, Bailey SR, Doherty JU, Douglas PS, Hendel RC, Kramer CM (2014). ACCF/AHA/ASE/ASNC/HFSA/HRS/SCAI/SCCT/SCMR/STS 2013 multimodality appropriate use criteria for the detection and risk assessment of stable ischemic heart disease: a report of the American College of Cardiology Foundation Appropriate Use Criteria Task Force, American Heart Association, American Society of Echocardiography, American Society of Nuclear Cardiology, Heart Failure Society of America, Heart Rhythm Society, Society for Cardiovascular Angiography and Interventions, Society of Cardiovascular Computed Tomography, Society for Cardiovascular Magnetic Resonance, and Society of Thoracic Surgeons. J Am Coll Cardiol.

[3] Gulati M, Levy PD, Mukherjee D, Amsterdam E, Bhatt DL, Birtcher KK (2021). 2021 AHA/ACC/ASE/CHEST/SAEM/SCCT/SCMR guideline for the evaluation and diagnosis of chest pain: a report of the American College of Cardiology/American Heart Association joint committee on clinical practice guidelines. Circulation.

[4] Knuuti J, Wijns W, Saraste A, Capodanno D, Barbato E, Funck-Brentano C (2020). 2019 ESC Guidelines for the diagnosis and management of chronic coronary syndromes. Eur Heart J.

[5] Greenwood JP, Maredia N, Younger JF, Brown JM, Nixon J, Everett CC (2012). Cardiovascular magnetic resonance and single-photon emission computed tomography for diagnosis of coronary heart disease (CE-MARC): a prospective trial. Lancet.

[6] Greenwood JP, Ripley DP, Berry C, McCann GP, Plein S, Bucciarelli-Ducci C (2016). Effect of care guided by cardiovascular magnetic resonance, myocardial perfusion scintigraphy, or NICE guidelines on subsequent unnecessary angiography rates: The CE-MARC 2 Randomized Clinical Trial. JAMA.

[7] Danad I, Szymonifka J, Twisk JWR, Norgaard BL, Zarins CK, Knaapen P (2017). Diagnostic performance of cardiac imaging methods to diagnose ischaemia-causing coronary artery disease when directly compared with fractional flow reserve as a reference standard: a meta-analysis. Eur Heart J.

[8] Juarez-Orozco LE, Saraste A, Capodanno D, Prescott E, Ballo H, Bax JJ (2019). Impact of a decreasing pre-test probability on the performance of diagnostic tests for coronary artery disease. Eur Heart J Cardiovasc Imaging.

[9] Ge Y, Pandya A, Steel K, Bingham S, Jerosch-Herold M, Chen YY (2020). Cost-effectiveness analysis of stress cardiovascular magnetic resonance imaging for stable chest pain syndromes. JACC Cardiovasc Imaging.

[10] Walker S, Girardin F, McKenna C, Ball SG, Nixon J, Plein S (2013). Cost-effectiveness of cardiovascular magnetic resonance in the diagnosis of coronary heart disease: an economic evaluation using data from the CE-MARC study. Heart.

[11] Kalb B, Indik JH, Ott P, Martin DR (2018). MRI of patients with implanted cardiac devices. J Magn Reson Imaging.

[12] Nazarian S, Hansford R, Rahsepar AA, Weltin V, McVeigh D, Gucuk Ipek E, Kwan A, Berger RD, Calkins H, Lardo AC, Kraut MA, Kamel IR, Zimmerman SL, Halperin HR (2017). Safety of magnetic resonance imaging in patients with cardiac devices. N Engl J Med.

[13] Wong ND, Budoff MJ, Ferdinand K, Graham IM, Michos ED, Reddy T (2022). Atherosclerotic cardiovascular disease risk assessment: an American Society for Preventive Cardiology clinical practice statement. Am J Prev Cardiol.

[14] Wagner A, Mahrholdt H, Holly TA, Elliott MD, Regenfus M, Parker M (2003). Contrast-enhanced MRI and routine single photon emission computed tomography (SPECT) perfusion imaging for detection of subendocardial myocardial infarcts: an imaging study. Lancet.

[15] Klein C, Nekolla SG, Bengel FM, Momose M, Sammer A, Haas F (2002). Assessment of myocardial viability with contrast-enhanced magnetic resonance imaging: comparison with positron emission tomography. Circulation.

[16] Izquierdo M, Ruiz-Granell R, Bonanad C, Chaustre F, Gomez C, Ferrero A (2013). Value of early cardiovascular magnetic resonance for the prediction of adverse arrhythmic cardiac events after a first noncomplicated ST-segment-elevation myocardial infarction. Circ Cardiovasc Imaging.

[17] Harries I, Liang K, Williams M, Berlot B, Biglino G, Lancellotti P (2020). Magnetic resonance imaging to detect cardiovascular effects of cancer therapy: JACC CardioOncology State-of-the-Art Review. JACC CardioOncol.

[18] Raman SV, Markl M, Patel AR, Bryant J, Allen BD, Plein S (2022). 30-minute CMR for common clinical indications: a Society for Cardiovascular Magnetic Resonance white paper. J Cardiovasc Magn Reson.

